# Cord blood DNA methylation and adiposity measures in early and mid-childhood

**DOI:** 10.1186/s13148-017-0384-9

**Published:** 2017-08-15

**Authors:** Jacob K. Kresovich, Yinan Zheng, Andres Cardenas, Brian T. Joyce, Sheryl L. Rifas-Shiman, Emily Oken, Matthew W. Gillman, Marie-France Hivert, Andrea A. Baccarelli, Lifang Hou

**Affiliations:** 1 Division of Epidemiology and Biostatistics, School of Public Health, University of Illinois at Chicago, Chicago, IL USA; 2 Center for Population Epigenetics, Robert H. Lurie Comprehensive Cancer Center and Department of Preventive Medicine, Northwestern University, Chicago, IL USA; 3 Division of Chronic Disease Research Across the Lifecourse (CoRAL), Department of Population Medicine, Harvard Medical School and Harvard Pilgrim Health Care Institute, Boston, MA USA; 4 Environmental Influences on Child Health Outcomes (ECHO) Program, Office of the Director, National Institutes of Health, Bethesda, MD USA; 5 Department of Environmental Health Science, Mailman School of Public Health, Columbia University, New York, NY USA

**Keywords:** EWAS, Cord blood, Methylation, Adiposity, Childhood adiposity

## Abstract

**Background:**

Excess adiposity in childhood is associated with numerous adverse health outcomes. As this condition is difficult to treat once present, identification of risk early in life can help inform and implement strategies to prevent the onset of the condition. We performed an epigenome-wide association study to prospectively investigate the relationship between cord blood DNA methylation and adiposity measurements in childhood.

**Methods:**

We measured genome-wide DNA methylation from 478 children in cord blood and measured overall and central adiposity via skinfold caliper measurements in early (range 3.1–3.3 years) and mid-childhood (age range 7.3–8.3 years) and via dual X-ray absorptiometry (DXA) in mid-childhood. Final models were adjusted for maternal age at enrollment, pre-pregnancy body mass index, education, folate intake during pregnancy, smoking during pregnancy, and gestational weight gain, and child sex, race/ethnicity, current age, and cord blood cell composition.

**Results:**

We identified four promoter proximal CpG sites that were associated with adiposity as measured by subscapular (SS) and triceps (TR) ratio (SS:TR) in early childhood, in the genes *KPRP*, *SCL9A10*, *MYLK2*, and *PRLHR*. We additionally identified one gene body CpG site associated with early childhood SS + TR on *PPAPDC1A*; this site was nominally associated with SS + TR in mid-childhood. Higher methylation at one promoter proximal CpG site in *MMP25* was also associated with SS:TR in mid-childhood. In regional analyses, methylation at an exonal region of *GFPT2* was positively associated with SS:TR in early childhood. Finally, we identified regions of two long, non-coding RNAs which were associated with SS:TR (LOC100049716) and fat-free mass index (LOC102723493) in mid-childhood.

**Conclusion:**

This analysis identified novel CpG loci associated with adiposity outcomes. However, our results suggest little consistency across the various adiposity outcomes tested, particularly among the more accurate DXA measurements of body composition. We recommend using caution when interpreting these associations.

**Electronic supplementary material:**

The online version of this article (doi:10.1186/s13148-017-0384-9) contains supplementary material, which is available to authorized users.

## Background

Nearly one third of American children are overweight or obese, and this proportion has tripled among adolescents and more than doubled in younger children since 1980 [1, 2]. Obesity and excess adiposity in childhood and adolescence are associated with adverse metabolic, orthopedic, cardiovascular, psychological, neurological, hepatic, pulmonary, and renal outcomes [3–7]. Currently, screening strategies for obesity and excess adiposity include tracking children’s body mass index (BMI) at pediatrician visits. This strategy may miss a proportion of children at risk for adverse health outcomes as BMI incorporates both fat and lean mass and does not indicate the distribution of fat, which has been shown to be a better predictor of metabolic health hazards [8, 9]. While excess adiposity itself is only a risk factor for future disease development, once present, it is difficult to treat. Therefore, the identification of risk early in life can help inform and implement strategies to prevent the onset of abnormal weight gain and the trajectory towards the development of excess adiposity.

A few studies have examined blood DNA methylation associations with adiposity- or obesity-related outcomes using cross-sectional and candidate gene approaches. Two cross-sectional studies employing an epigenome-wide approach in blood leucocytes identified CpG sites at which DNA methylation was associated with obesity status and BMI percentile [10, 11]. An additional study using a prospective, candidate gene approach showed methylation at chr9:136355885 (hg18) of *RXRA* measured in cord blood was associated with fat mass and percent fat mass at age 9 [12]. Together, these findings suggest that blood DNA methylation may serve as an additional screening tool to help identify children at risk for developing cardiometabolic risk factors in childhood. Conversely, DNA methylation marks associated with adiposity might be a consequence rather than a cause as suggested by recent analyses of large epidemiological studies [13].

Cord blood DNA methylation offers a unique screening opportunity as it is established in utero and may mediate associations of maternal exposures, such as maternal smoking and gestational weight gain, with childhood obesity [14–18]. Few epigenome-wide studies have prospectively investigated the relationship between cord blood methylation and adiposity-related measurements in childhood [19]. The purpose of this study was to examine associations of cord blood DNA methylation patterns with various measures of adiposity in early and mid-childhood in the Project Viva cohort. We hypothesized that cord blood DNA methylation could serve as a predictive biomarker of childhood adiposity.

## Methods

### Study population

The children for our study were participants in Project Viva, a pre-birth cohort conducted in eastern Massachusetts, USA [20]. Briefly, we recruited pregnant women between 1999 and 2002 during their first prenatal visit at Atrius Harvard Vanguard Medial Associates, a large multispecialty practice. Eligibility criteria were as follows: fluency in English, gestational age less than 22 weeks, and singleton pregnancy. At recruitment, mothers self-reported their height and pre-pregnancy weight, which we used to calculate pre-pregnancy BMI. We collected maternal diet and behaviors via validated questionnaires [21]. Additional details of the cohort are published elsewhere [20]. Of the 2128 mother-infant pairs, DNA methylation was measured in cord blood from 507 children. Of these, 22 samples were excluded due to low sample quality leaving 485 samples. A total of 478 offspring had cord blood DNA methylation measurements and at least one measure of adiposity in early or mid-childhood; 460 had complete covariate data (missing data were not imputed), of which 415 had early childhood skinfold measurements, 402 had mid-childhood skinfold measurements, and 319 had mid-childhood dual X-ray absorptiometry (DXA) measurements.

### Adiposity measurements

In early and mid-childhood visits, subscapular (SS) and triceps (TR) skinfold thicknesses were measured to the nearest 0.1 mm using Holtain calipers (Holtain Ltd., Crosswell, Wales); height was measured to the nearest 0.1 cm using a calibrated stadiometer (Shorr Productions, Olney, MD), and weight was measured to the nearest 0.1 kg using a calibrated scale (Tanita model TBF-300A, Tanita Corporation of America, Inc., Arlington Heights, IL). In mid-childhood, trained research assistants performed whole body DXA scans on the children using a Hologic model Discovery A (Hologic, Bedford, MA) that was checked for quality control daily by scanning a standard synthetic spine to examine measurement drift. Measures of adiposity were calculated using Hologic software QDR version 12.6. All scans were examined for positioning, movement, and artifacts by a single trained investigator. The same researcher identified defined body regions for analyses. Intra-rater reliability on duplicate measurements was high (*r* = 0.99) [22].

We calculated adiposity outcomes in early and mid-childhood. For both time points, we combined SS and TR measurements in two ways: (1) sum of skinfold thickness (SS + TR) to represent overall adiposity and (2) ratio of skinfold thickness (SS:TR) to represent central adiposity. In mid-childhood, we used DXA measurements to calculate measures of overall adiposity (total fat mass index (kg/m^2^), fat-free mass index (kg/m^2^), and total percent fat (%)) and central adiposity (truncal fat mass index (kg/m^2^)).

### Cord blood DNA methylation measurements

Trained medical personnel collected venous umbilical cord blood samples immediately upon delivery. Samples were stored at 4 °C and transported to a central location for processing within 24 h of collection. Buffy coat DNA was extracted on the day of arrival using the Qiagen Puregene Kit (Valencia, CA). Extracted DNA aliquots were stored at − 80 °C until analysis.

We converted DNA with sodium bisulfate using the EZ DNA Methylation-Gold Kit (Zymo Research, Irving, CA). We provided samples to Illumina, Inc., for analyses using the Infinium HumanMethylation450 BeadChip (Illumina, San Diego, CA) following standard manufacturer’s protocols. Raw methylation image files were processed using the *minfi* package in *R* [23]. Correlation coefficients for individual probes among all technical replicates ranged from 0.98 to 1. Individual probes were excluded if they had non-significant *p* values for detection in greater than 5% of the samples. We additionally excluded CpG probes on sex chromosomes. Single-nucleotide polymorphism (SNP)-associated probes were removed for SNPs with a minor-allele frequency of ≥ 5%. Furthermore, we removed previously identified non-specific and cross-reactive probes within the array along with polymorphic CpG loci [24, 25]. Background correction and dye-bias equalization was performed via the normal-exponential out-of-band (*noob*) correction method [26]. We additionally applied a β-mixture quantile intra sample normalization procedure (BMIQ) to minimize potential probe-type bias. Finally, ComBat was used to correct for batch effects from plate and protect against regressing variability due to covariates [27]. After quality control, the total number of probes left for analysis was 372,563.

### Statistical analysis

We calculated means and standard deviations (SD) or median and interquartile ranges for all maternal and child characteristics to describe the study population overall and stratified by child sex. Adiposity outcomes were skewed, and therefore, we log-transformed them. We elected to include child sex, race/ethnicity, and current age as *a priori* covariates in all analyses. We additionally examined maternal pre-pregnancy BMI, age at enrollment, parity, education, mean folate and vitamin B intake during pregnancy, gestational weight gain, gestational diabetes status, smoking during pregnancy, and mode of delivery as potential confounders. Maternal covariates were added to the final models if they were associated with any of the log-transformed adiposity outcomes in linear regression models at *p* < 0.05. Because cord blood cell composition is related to methylation, we estimated blood sample cell proportions of CD8+, CD4+, natural killer cells, monocytes, granulocytes, B cells, and nucleated red blood cells based on the Bakulski et al. reference panel [28]. The final models were adjusted for maternal pre-pregnancy BMI, age at enrollment, education, mean folate intake during pregnancy, smoking during pregnancy, and gestational weight gain, and child sex, race/ethnicity, current age, and cord blood cell composition. All analyses were conducted on individuals with complete covariate information; we additionally conducted a sensitivity analysis examining the influence of adjusting for child birth weight in our epigenome-wide association study analyses.

#### Epigenome-wide association study (EWAS)

Single CpG site percent methylation beta values (*β*) were converted to *M* values using a logit transformation [29]. We use *M* values in statistical analysis to minimize heteroscedasticity in regression models allowing for a more precise and valid measurement of associations. Using multiple linear regression models, we examined associations between individual CpG sites in cord blood and adiposity measurements from both early and mid-childhood. Statistical significance for genome-wide associations were adjusted for multiple comparisons using a false discovery rate (FDR) *q* < 0.05.

#### Regional analyses

As previous studies have identified highly correlated methylation values among neighboring CpG sites [30], we explored regional genomic associations with adiposity outcomes. We examined the association of childhood adiposity with differentially methylated regions (DMRs) in cord blood using the *R* Bioconductor package Bumphunter [31]. Briefly, Bumphunter determines candidate DMRs based on a resampling procedure while adjusting for covariates. Minimum number of probes per region were set at two with a max distance of 1000 base pairs. Loess smoothing was applied to each genomic cluster. Results were bootstrapped 1000 times to generate null candidate regions. Significant testing among DMRs were adjusted for multiple comparisons using family-wise error rate (FWER) < 0.05.

## Results

 Descriptive statistics of the study population are shown overall and stratified by offspring sex in Table 1. Mothers had a mean age of 32.1 (SD = 5.3) at enrollment, nearly half (46%) were nulliparous, most were well educated (66% completed college), and most (68%) had never smoked in their lifetime. During pregnancy, the mothers gained an average of 15.5 kg (SD = 5.5) and only a small proportion smoked (11%). The offspring were majority white (67%) and approximately half female (48%).

**Table 1:** Characteristics of mothers and offspring with cord blood measurements

	Total	Girls	Boys
	*n* = 478	*n* = 229	*n* = 249
	Mean (SD) or *N* (%)		
Mother			
Maternal age at enrollment, years	32.1 (5.3)	32.6 (4.9)	31.6 (5.6)
Nulliparous: *N* (%)			
No	257 (53.8)	123 (53.7)	134 (53.8)
Yes	221 (46.2)	106 (46.3)	115 (46.2)
College graduate: *N* (%)			
No	160 (33.5)	75 (32.8)	85 (34.1)
Yes	318 (66.5)	154 (67.2)	164 (65.9)
Smoking status: *N* (%)			
Never	325 (68.0)	161 (70.3)	164 (65.9)
Former	100 (20.9)	50 (21.8)	50 (20.1)
During pregnancy	53 (11.1)	18 (7.9)	35 (14.1)
Pre-pregnancy BMI, kg/m^2^	24.8 (5.3)	24.3 (5.0)	25.2 (5.6)
Gestational weight gain, kg	15.5 (5.5)	15.1 (5.3)	15.8 (5.6)
Folate mean, μg	1071 (334)	1097 (313)	1046 (351)
Vitamin B12 mean, μg	10.2 (4.5)	10.3 (4.5)	10.2 (4.6)
Child			
Race/ethnicity: *N* (%)			
Black	60 (12.6)	27 (11.8)	33 (13.3)
Hispanic	25 (5.2)	9 (3.9)	16 (6.4)
White	322 (67.4)	162 (70.7)	160 (64.3)
Other	71 (14.9)	31 (13.5)	40 (16.1)
Gestation length, weeks	39.7 (1.6)	39.8 (1.6)	39.6 (1.7)
*Early childhood visit*			
Age, years: median (IQR)	3.2 (3.1–3.3)	3.1 (3.1–3.3)	3.2 (3.1–3.3)
BMI z-score: median (IQR)	0.45 (− 0.26 - 1.10)	0.39 (− 0.23 - 1.08)	0.49 (− 0.29 - 1.11)
Waist circumference, cm: median (IQR)	50.8 (48.6–53.3)	50.6 (48.5–53.4)	51.0 (48.6–53.0)
SS + TR, mm: median (IQR)	16.0 (13.6–18.8)	17.0 (14.4–20.0)	15.2 (13.4–17.8)
SS:TR ratio: median (IQR)	0.61 (0.53–0.72)	0.63 (0.51–0.73)	0.60 (0.54–0.72)
*Mid-childhood visit*			
Age, years: median (IQR)	7.7 (7.3–8.3)	7.6 (7.3–8.2)	7.7 (7.3–8.4)
Waist circumference, cm: median (IQR)	57.7 (54.3–62.1)	57.6 (53.9–62.5)	57.7 (55.0–61.9)
SS + TR, mm: median (IQR)	16.4 (13.2–21.4)	17.8 (14.2–22.8)	15.2 (12.8–19.6)
SS:TR ratio: median (IQR)	0.65 (0.56–0.79)	0.64 (0.55–0.79)	0.66 (0.57–0.78)
DXA total fat mass index, kg/m^2^: median (IQR)	3.8 (3.1–5.0)	4.2 (3.4–5.5)	3.5 (2.9–4.6)
DXA fat-free mass index, kg/m^2^: median (IQR)	12.7 (12.0–13.8)	12.4 (11.5–13.3)	13.3 (12.5–14.0)
DXA truncal fat mass index, kg/m^2^: median (IQR)	1.2 (0.9–1.6)	1.3 (1.0–1.9)	1.0 (0.9–1.5)
DXA total percent fat: median (IQR)	23.0 (19.9–27.6)	25.8 (21.8–29.6)	21.2 (18.6–25.4)

*DXA* dual X-ray absorptiometry, *IQR* interquartile range, *SS* subscapular, *TR* triceps

**Fig. 1:**
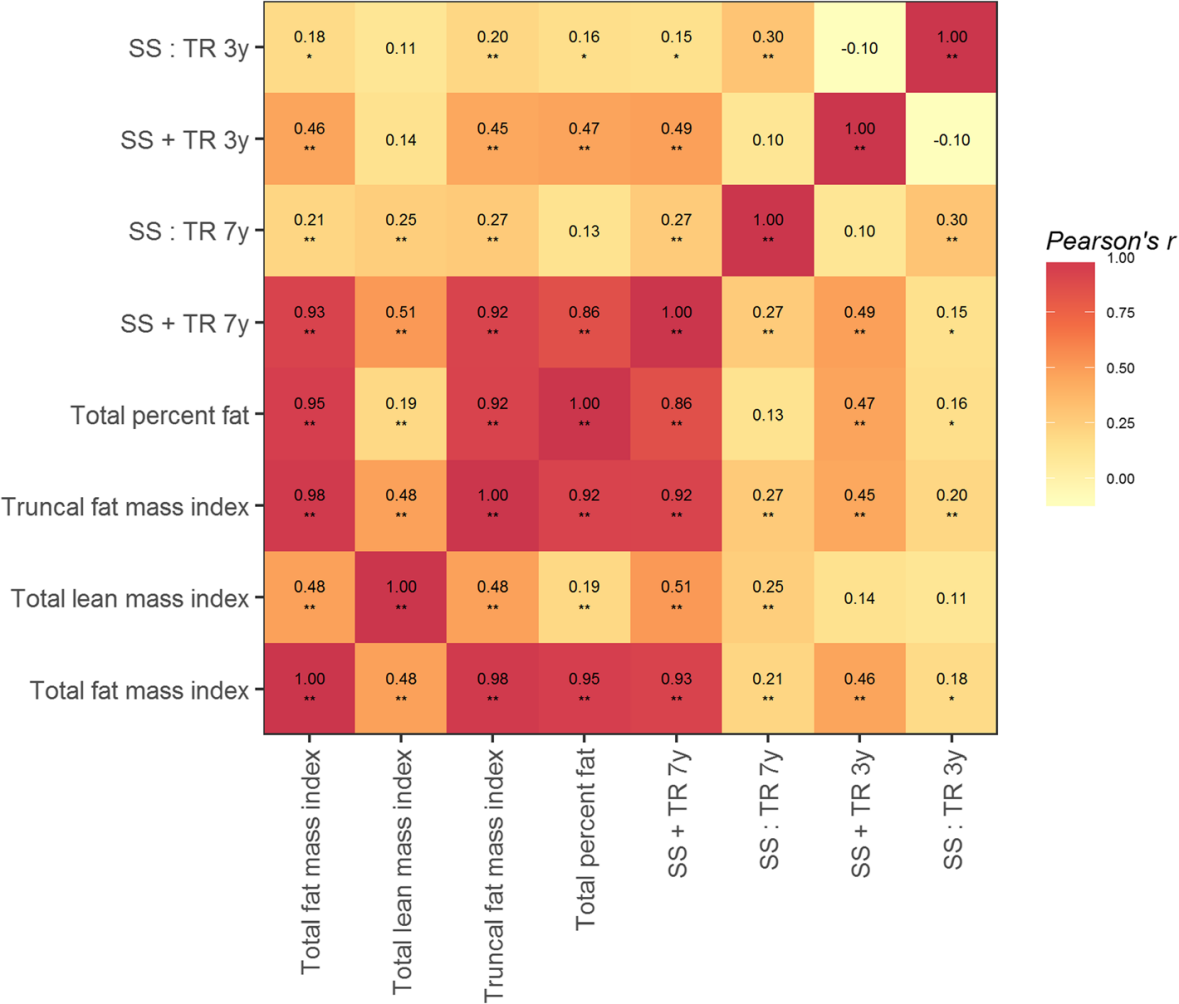
Pearson’s correlation matrix of the log-transformed adiposity outcomes measured in early and mid-childhood

Figure 1 depicts a matrix showing the correlations across the log-transformed adiposity outcomes of interest. Generally, with the exception of total lean mass index, the DXA measurements obtained in mid-childhood were strongly correlated with one another. Total fat mass index was highly correlated with both truncal fat mass index (Pearson’s *r* (*ρ* = 0.98)) and total percent fat (*ρ* = 0.95). Additionally, SS + TR measured in mid-childhood was strongly correlated with total fat mass index (*ρ* = 0.93), truncal fat mass index (*ρ* = 0.92), and total percent fat (*ρ* = 0.86). Early childhood SS:TR and SS + TR were moderately correlated with measurements taken in mid-childhood (*ρ* = 0.30, *ρ* = 0.49, respectively).

**Fig. 2:**
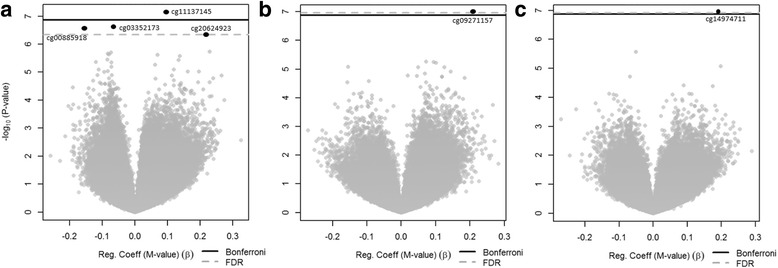
Volcano plots of the associations between cord blood DNA methylation and early childhood measurements of SS:TR (**a**), SS + TR (**b**), and mid-childhood measurements of SS:TR (**c**). Significant loci are located above the *gray dotted line*, representing an FDR cut point of 0.05

Figure 2 depicts volcano plots showing the epigenome-wide associations between cord blood DNA methylation and adiposity outcomes. Figure 2a shows that methylation at cg11137145 (*β* = 0.10, 95% CI 0.06, 0.13, FDR *q* = 0.02), cg03352173 (*β* = − 0.07, 95% CI − 0.09, − 0.04, FDR *q* = 0.03), cg00885918 (*β* = −0.15, 95% CI − 0.21, − 0.09, FDR *q* = 0.03), and cg20624923 (*β* = 0.22, 95% CI 0.14, 0.31, FDR *q* = 0.03) met our significance threshold for SS:TR measured in early childhood. These four CpG sites are located in the promoter regions of *KPRP* (chr1: 152,730,027), *SLC9A10* (chr3: 112,013,130), *MYLK2* (chr20: 30,406,997), and *PRLHR* (chr10: 120,355,428), respectively. Figure 2b shows that higher methylation at cg09271157 (*β* = 0.21, 95% CI 0.13, 0.28, FDR *q* = 0.04) was significantly associated with greater SS + TR measured in early childhood. This CpG site is located in the gene body of *PPAPDC1A* (chr10: 122,217,376). Finally, Fig. 2c shows that higher methylation of cg14974711 (*β* = 0.19, 95% CI 0.12, 0.26, FDR *q* = 0.02) was significantly associated with greater SS:TR measured in mid-childhood. This CpG site is located in the promoter region of *MMP25* (chr16: 3,096,478). No individual CpG sites met genome-wide significance for mid-childhood SS + TR, or any of the DXA measurements. In sensitivity analyses, we identified the same CpG sites with similar associations when additionally adjusting for child birth weight (Additional file 1: Table S1). Finally, in order to explore persistence of these associations across follow-up visits, we examined the relationships between the identified CpG sites with the caliper measures (SS + TR and SS:TR) in both early and mid-childhood (Additional file 1: Table S2); across follow-up visits, associations were generally attenuated. Although no CpG sites showed significance at *q* < 0.05 at both follow-up visits, cg09271157 of *PPAPDC1A* (identified as associated with SS + TR measured in early childhood) was nominally associated with SS + TR in mid-childhood (*p* = 0.05).

**Fig. 3:**
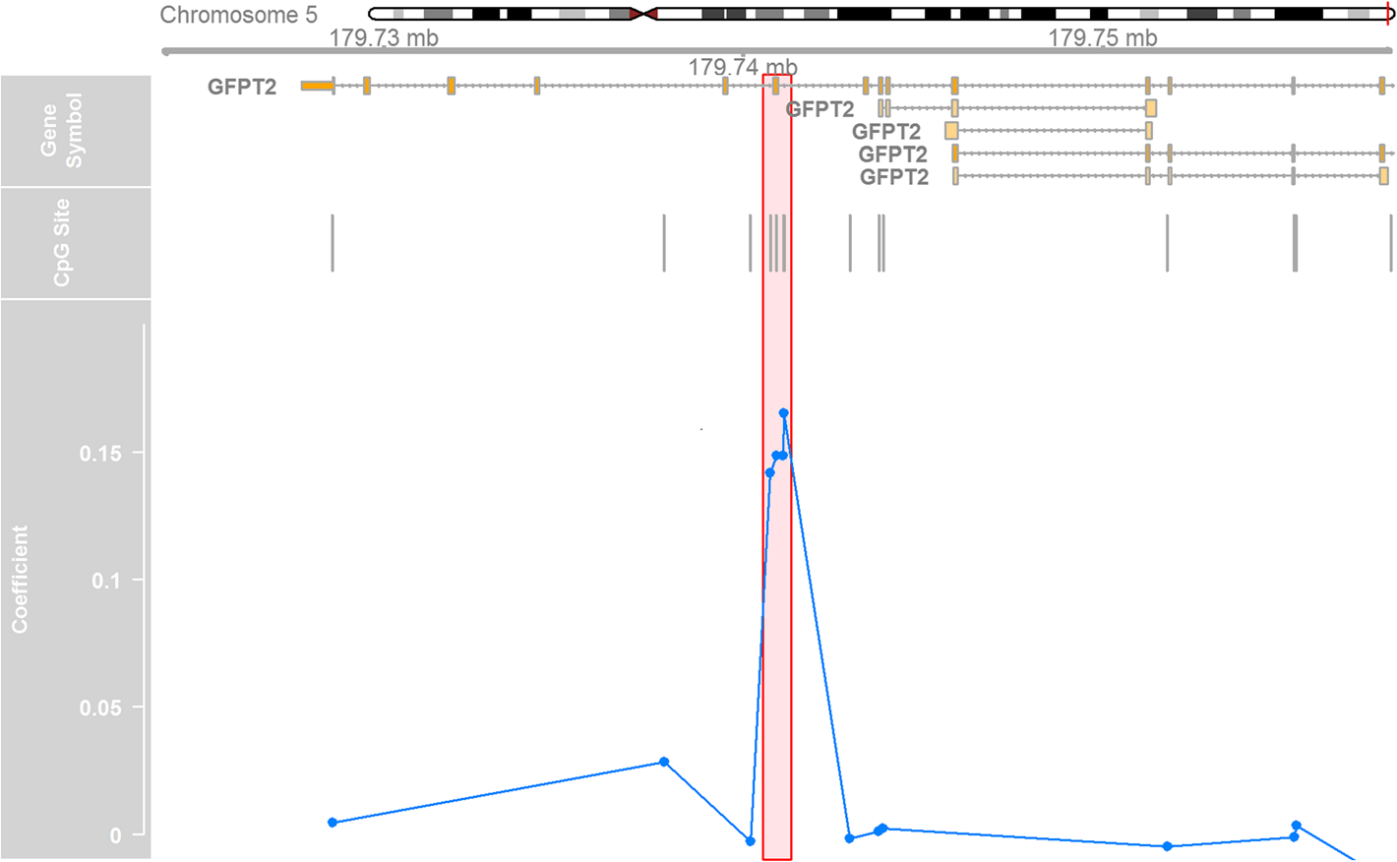
Chromosomal, genomic, and CpG site locations of identified regional association with SS:TR in early childhood. *Highlighted box* shows methylation of exon 14 of *GFPT2* was positively associated with SS:TR in early childhood. Four CpG sites (cg23221052, cg13944838, cg23248424, and cg02891314) showed higher methylation (denoted by positive coefficients) in offspring with greater SS:TR values

In regional analyses, we identified one region that met FWER < 0.05 for early childhood adiposity. Figure 3 depicts a region of four CpG sites (cg23221052, cg13944838, cg23248424, and cg02891314) which was positively associated with SS:TR, indicating that higher methylation levels at this region was associated with greater central adiposity. The region is located in exon 14 of *GFPT2* (chr5: 179,740,743–179,741,120; FWER = 0.02). Finally, we identified regions for two long, non-coding RNAs which were associated with SS:TR (LOC100049716) and fat-free mass index (LOC102723493) in mid-childhood.

## Discussion

In this study, we observed that methylation at individual cord blood CpG loci from *PRLHR*, *KPRP*, *SLC9A10*, *MYLK2*, and *PPAPDC1A*, as well as an exonal region of *GFPT2*, were associated with various measures of adiposity in early childhood. We additionally identified an association between single-site *MMP25* methylation and central adiposity, as well as regional associations between long, non-coding RNAs with measures of adiposity in mid-childhood. While these results identified novel loci and regions associated with adiposity outcomes throughout childhood, our results suggest little consistency across the various adiposity outcomes tested, particularly among the more accurate DXA measurements of body composition.

We identified multiple sites in promoter proximal regions associated with the SS:TR measured in early childhood, namely *PRLHR*, *KPRP*, *MYLK2*, and *SLC9A10*. While there exists no evidence linking *KPRP*, *MYLK2*, and *SLC9A10* to adiposity outcomes, our findings for *PRLHR* are supported by previous studies. *PRLHR* encodes a transmembrane protein for the prolactin-releasing hormone and has previously been associated with body weight control and obesity [32–37]. This gene is mainly expressed in the reticular nucleus of the thalamus [38] and controls metabolic rate and appetite by acting as a receptor for the stimulation of prolactin release [39]. Ding et al. [34] identified and validated an association between promoter methylation of *PRLHR* and childhood obesity in a population of Chinese preschool children. While this previous study was cross-sectional in nature, the prospective design of our study suggests *PRLHR* methylation as an event prior to the development of central adiposity.

Using early childhood measurements, we also identified a regional association between exon 14 of *GFPT2* and SS:TR. *GFPT2* encodes a protein that controls the flux of glucose into the hexosamine pathway. While no human or animal studies implicate *GFPT2* methylation in the development of excess adiposity, genome-wide association studies identified an association between a SNP (rs2303007) in exon 14 of *GFTP2* with development of type 2 diabetes in Caucasian individuals [40, 41], although this SNP failed replication in a candidate SNP study of a Finnish population [42]. Additionally, a SNP (rs10479469) located approximately 32-kb downstream of *GFPT2* was associated with arm subcutaneous adipose tissue in HIV-infected men [43]. Our finding adds to the growing literature linking *GFTP2* with adiposity-related outcomes. The proposed mechanism involves chronically increased expression of *GFPT2* messenger RNA (mRNA) resulting in increased hexosamine flux. In our study, we showed higher methylation of the *GFPT2* gene body was associated with greater central adiposity. While promoter hypermethylation of *GFPT2* is associated with decreased circulating levels of *GFPT2* mRNA [44], the association between *GFPT2* gene body methylation and its expression is unknown, although studies have found positive associations between gene body methylation and expression in other genes [45, 46]. These results suggest a potential link to regulation of adipose tissue distribution, although more research is needed to support these findings.

To examine persistence of these associations across follow-up visits, we examined associations between cord blood DNA methylation of these identified CpG sites with caliper measures in mid-childhood and generally saw an attenuation of the estimates across follow-up visits. We showed that methylation at birth at cg09271157 of the gene body of *PPAPDC1A* was significantly associated with SS + TR in early childhood at *q* < 0.05 and in mid-childhood at a nominal *p* < 0.05. While *PPAPDC1A* has never been implicated in the development of adiposity, it may serve as a predictive biomarker of future adiposity-related outcomes.

Regarding our analyses using mid-childhood adiposity outcomes, we identified an association between *MMP25* methylation and central adiposity as represented by SS:TR measurements. *MMP25* encodes a protein in the membrane-type subfamily of matrix metalloproteinases which is commonly attached to the plasma membrane in an inactive state [47]. MMP25 is activated when cleaved by extracellular proteinases in response to bacterial infection or inflammation and is believed to inactivate the alpha-1 proteinase inhibitor. Although no previous human or animal studies have implicated *MMP25* in the development of obesity, decreased levels of circulating alpha-1 proteinase inhibitor were associated with increased BMI in Chinese men [48]. Future studies are warranted to better elucidate this pathway or potential use as a biomarker of obesity risk.

While this study has novel findings, there are a number of limitations worth mentioning. Most importantly, this study examined associations between cord blood DNA methylation and six adiposity measurements taken in mid-childhood (two of which were additionally measured in early childhood). While we did correct for multiple comparisons examining individual CpG sites and genomic regions within adiposity phenotypes using FDR and FWER <0.05, we did not account for multiple testing across outcomes as we consider these independent hypotheses. This is particularly important because the cord blood methylation associations with different measures of overall adiposity differed greatly, particularly among the highly correlated DXA measurements. Furthermore, there is little evidence to support the role of *KPRP*, *MYLK2*, *SLC9A10*, *PPAPDC1A*, and *MMP25* methylation with the development of excess adiposity. Although importantly, our finding linking *PRLHR* promoter methylation with SS:TR builds upon the existing literature implicating this gene in development of excess adiposity. This study was also likely underpowered for the number of analyses undertaken due to the low amount of variability in our examined outcomes. We attempted to address this issue by adjusting for variables that strongly predict adiposity (e.g., current child age) in order to increase estimate precision. One possible explanation for our lack of consistent findings is that previous studies that have shown changes in methylation are often a consequence of adiposity, rather than a cause of it [13, 49]. The limited number of significant CpG sites where methylation levels in cord blood are associated with adiposity later in childhood suggests that previous cross-sectional reports of adiposity association with DNA methylation may be affected by reverse causation. While we did identify some novel loci associated with skinfold measurements, additional studies in larger cohorts will be necessary to add validity to our findings.

## Conclusion

This was the first study of which we are aware to use an epigenome-wide approach to examine the association between cord blood DNA methylation in the development of excess adiposity phenotypes through mid-childhood using objective measurements. Excess adiposity is difficult to treat once present; therefore, identification of biomarkers of risk early in life would be a valuable tool to help target individuals who would benefit from particular attention to lifestyle factors to prevent excess weight gain. While this analysis did identify novel CpG sites and regions of the genome statistically associated with adiposity outcomes, particularly *PRLHR*, due to the lack of concordance across similar outcomes, we recommend using caution when interpreting these associations. Future studies should continue to examine cord blood DNA methylation as a predictor of childhood obesity and excess adiposity but explore this research question in a larger, more representative population.

## Additional file

Additional file 1: Online supplemental tables. (DOCX 18 kb)

## Abbreviations

BMI: Body mass index
DMRs: Differentially methylated regions
DXA: Dual X-ray absorptiometry
EWAS: Epigenome-wide association study
FDR: False discovery rate
SD: Standard deviations
SNP: Single-nucleotide polymorphism
SS + TR: Sum of skinfold thickness
SS: Subscapular
SS:TR: Ratio of skinfold thickness
TR: Triceps

## References

[1] Ogden CL, Carroll MD, Kit BK, Flegal KM (2012). Prevalence of obesity and trends in body mass index among US children and adolescents, 1999-2010. JAMA.

[2] National Center for Health Statistics (2007). Health, United States, 2007 with chartbook on trends in the health of Americans.

[3] Daniels SR, Arnett DK, Eckel RH, Gidding SS, Hayman LL, Kumanyika S, Robinson TN, Scott BJ, St Jeor S, Williams CL (2005). Overweight in children and adolescents: pathophysiology, consequences, prevention, and treatment. Circulation.

[4] Dietz WH (1998). Health consequences of obesity in youth: childhood predictors of adult disease. Pediatrics.

[5] Must A, Jacques PF, Dallal GE, Bajema CJ, Dietz WH (1992). Long-term morbidity and mortality of overweight adolescents. A follow-up of the Harvard growth study of 1922 to 1935. N Engl J Med.

[6] Must A, Strauss RS (1999). Risks and consequences of childhood and adolescent obesity. Int J Obes Relat Metab Disord.

[7] Freedman DS, Dietz WH, Srinivasan SR, Berenson GS (1999). The relation of overweight to cardiovascular risk factors among children and adolescents: the Bogalusa Heart Study. Pediatrics.

[8] Després JP (2006). Is visceral obesity the cause of the metabolic syndrome?. Ann Med.

[9] Després JP, Lemieux I (2006). Abdominal obesity and metabolic syndrome. Nature.

[10] Wang X, Zhu H, Snieder H, Su S, Munn D, Harshfield G, Maria BL, Dong Y, Treiber F, Gutin B, Shi H (2010). Obesity related methylation changes in DNA of peripheral blood leukocytes. BMC Med.

[11] Ali O, Cerjak D, Kent JW, James R, Blangero J, Carless MA, Zhang Y. Methylation of SOCS3 is inversely associated with metabolic syndrome in an epigenome-wide association study of obesity. Epigenetics. 2016;11(9):699–707. 10.1080/15592294.2016.1216284 PMC5048720 27564309

[12] Godfrey KM, Sheppard A, Gluckman PD, Lillycrop KA, Burdge GC, McLean C, Rodford J, Slater-Jefferies JL, Garratt E, Crozier SR (2011). Epigenetic gene promoter methylation at birth is associated with child’s later adiposity. Diabetes.

[13] Wahl S, Drong A, Lehne B, Loh M, Scott WR, Kunze S, Tsai PC, Ried JS, Zhang W, Yang Y (2017). Epigenome-wide association study of body mass index, and the adverse outcomes of adiposity. Nature.

[14] Heijmans BT, Tobi EW, Stein AD, Putter H, Blauw GJ, Susser ES, Slagboom PE, Lumey LH (2008). Persistent epigenetic differences associated with prenatal exposure to famine in humans. Proc Natl Acad Sci U S A.

[15] Tobi EW, Lumey LH, Talens RP, Kremer D, Putter H, Stein AD, Slagboom PE, Heijmans BT (2009). DNA methylation differences after exposure to prenatal famine are common and timing- and sex-specific. Hum Mol Genet.

[16] Talens RP, Jukema JW, Trompet S, Kremer D, Westendorp RG, Lumey LH, Sattar N, Putter H, Slagboom PE, Heijmans BT, Group P (2012). Hypermethylation at loci sensitive to the prenatal environment is associated with increased incidence of myocardial infarction. Int J Epidemiol.

[17] Oken E, Huh SY, Taveras EM, Rich-Edwards JW, Gillman MW (2005). Associations of maternal prenatal smoking with child adiposity and blood pressure. Obes Res.

[18] Oken E, Taveras EM, Kleinman KP, Rich-Edwards JW, Gillman MW (2007). Gestational weight gain and child adiposity at age 3 years. Am J Obstet Gynecol.

[19] Lin X, Lim IY, Wu Y, Teh AL, Chen L, Aris IM, Soh SE, Tint MT, MacIsaac JL, Morin AM (2017). Developmental pathways to adiposity begin before birth and are influenced by genotype, prenatal environment and epigenome. BMC Med.

[20] Oken E, Baccarelli AA, Gold DR, Kleinman KP, Litonjua AA, De Meo D, Rich-Edwards JW, Rifas-Shiman SL, Sagiv S, Taveras EM (2015). Cohort profile: project viva. Int J Epidemiol.

[21] Rifas-Shiman SL, Rich-Edwards JW, Kleinman KP, Oken E, Gillman MW (2009). Dietary quality during pregnancy varies by maternal characteristics in Project Viva: a US cohort. J Am Diet Assoc.

[22] Boeke CE, Oken E, Kleinman KP, Rifas-Shiman SL, Taveras EM, Gillman MW (2013). Correlations among adiposity measures in school-aged children. BMC Pediatr.

[23] Aryee MJ, Jaffe AE, Corrada-Bravo H, Ladd-Acosta C, Feinberg AP, Hansen KD, Irizarry RA (2014). Minfi: a flexible and comprehensive Bioconductor package for the analysis of Infinium DNA methylation microarrays. Bioinformatics.

[24] Chen YA, Lemire M, Choufani S, Butcher DT, Grafodatskaya D, Zanke BW, Gallinger S, Hudson TJ, Weksberg R (2013). Discovery of cross-reactive probes and polymorphic CpGs in the Illumina Infinium HumanMethylation450 microarray. Epigenetics.

[25] Zhang X, Mu W, Zhang W (2012). On the analysis of the Illumina 450 K array data: probes ambiguously mapped to the human genome. Front Genet.

[26] Triche TJ, Weisenberger DJ, Van Den Berg D, Laird PW, Siegmund KD (2013). Low-level processing of Illumina Infinium DNA Methylation BeadArrays. Nucleic Acids Res.

[27] Johnson WE, Li C, Rabinovic A (2007). Adjusting batch effects in microarray expression data using empirical Bayes methods. Biostatistics.

[28] Bakulski KM, Feinberg JI, Andrews SV, Yang J, Brown S, LMcKenney S, Witter F, Walston J, Feinberg AP, Fallin MD (2016). DNA methylation of cord blood cell types: applications for mixed cell birth studies. Epigenetics.

[29] Du P, Zhang X, Huang CC, Jafari N, Kibbe WA, Hou L, Lin SM (2010). Comparison of Beta-value and M-value methods for quantifying methylation levels by microarray analysis. BMC Bioinf.

[30] Eckhardt F, Lewin J, Cortese R, Rakyan VK, Attwood J, Burger M, Burton J, Cox TV, Davies R, Down TA, et al. DNA methylation profiling of human chromosomes 6, 20 and 22. Nat Genet. 2006;38:1378–85. 10.1038/ng1909 PMC3082778 17072317

[31] Jaffe AE, Murakami P, Lee H, Leek JT, Fallin MD, Feinberg AP, Irizarry RA. Bump hunting to identify differentially methylated regions in epigenetic epidemiology studies. Int J Epidemiol. 2012;41:200–9. 10.1093/ije/dyr238 PMC3304533 22422453

[32] Omori Y, Chaya T, Yoshida S, Irie S, Tsujii T, Furukawa T (2015). Identification of G protein-coupled receptors (GPCRs) in primary cilia and their possible involvement in body weight control. PLoS One.

[33] Gu W, Geddes BJ, Zhang C, Foley KP, Stricker-Krongrad A (2004). The prolactin-releasing peptide receptor (GPR10) regulates body weight homeostasis in mice. J Mol Neurosci.

[34] Ding X, Zheng D, Fan C, Liu Z, Dong H, Lu Y, Qi K (2015). Genome-wide screen of DNA methylation identifies novel markers in childhood obesity. Gene.

[35] Bjursell M, Lennerås M, Göransson M, Elmgren A, Bohlooly-Y M (2007). GPR10 deficiency in mice results in altered energy expenditure and obesity. Biochem Biophys Res Commun.

[36] Chan YF, Jones FC, McConnell E, Bryk J, Bünger L, Tautz D (2012). Parallel selection mapping using artificially selected mice reveals body weight control loci. Curr Biol.

[37] Yamakawa K, Kudo K, Kanba S, Arita J (1999). Distribution of prolactin-releasing peptide-immunoreactive neurons in the rat hypothalamus. Neurosci Lett.

[38] Hinuma S, Habata Y, Fujii R, Kawamata Y, Hosoya M, Fukusumi S, Kitada C, Masuo Y, Asano T, Matsumoto H (1998). A prolactin-releasing peptide in the brain. Nature.

[39] Zhang H, Jia Y, Cooper JJ, Hale T, Zhang Z, Elbein SC (2004). Common variants in glutamine: fructose-6-phosphate amidotransferase 2 (GFPT2) gene are associated with type 2 diabetes, diabetic nephropathy, and increased GFPT2 mRNA levels. J Clin Endocrinol Metab.

[40] Reynisdottir I, Thorleifsson G, Benediktsson R, Sigurdsson G, Emilsson V, Einarsdottir AS, Hjorleifsdottir EE, Orlygsdottir GT, Bjornsdottir GT, Saemundsdottir J (2003). Localization of a susceptibility gene for type 2 diabetes to chromosome 5q34-q35.2. Am J Hum Genet.

[41] Willer CJ, Bonnycastle LL, Conneely KN, Duren WL, Jackson AU, Scott LJ, Narisu N, Chines PS, Skol A, Stringham HM (2007). Screening of 134 single nucleotide polymorphisms (SNPs) previously associated with type 2 diabetes replicates association with 12 SNPs in nine genes. Diabetes.

[42] Irvin MR, Shrestha S, Chen YD, Wiener HW, Haritunians T, Vaughan LK, Tiwari HK, Taylor KD, Scherzer R, Saag MS (2011). Genes linked to energy metabolism and immunoregulatory mechanisms are associated with subcutaneous adipose tissue distribution in HIV-infected men. Pharmacogenet Genomics.

[43] Kuang SQ, Tong WG, Yang H, Lin W, Lee MK, Fang ZH, Wei Y, Jelinek J, Issa JP, Garcia-Manero G (2008). Genome-wide identification of aberrantly methylated promoter associated CpG islands in acute lymphocytic leukemia. Leukemia.

[44] Jones PA (2012). Functions of DNA methylation: islands, start sites, gene bodies and beyond. Nat Rev Genet.

[45] Rauscher GH, Kresovich JK, Poulin M, Yan L, Macias V, Mahmoud AM, Al-Alem U, Kajdacsy-Balla A, Wiley EL, Tonetti D, Ehrlich M (2015). Exploring DNA methylation changes in promoter, intragenic, and intergenic regions as early and late events in breast cancer formation. BMC Cancer.

[46] Flanagan JM, Brook MN, Orr N, Tomczyk K, Coulson P, Fletcher O, Jones ME, Schoemaker MJ, Ashworth A, Swerdlow A (2015). Temporal stability and determinants of white blood cell DNA methylation in the breakthrough generations study. Cancer Epidemiol Biomark Prev.

[47] English WR, Velasco G, Stracke JO, Knäuper V, Murphy G (2001). Catalytic activities of membrane-type 6 matrix metalloproteinase (MMP25). FEBS Lett.

[48] Xue GB, Zheng WL, Wang LH, Lu LY (2013). Alpha 1-antitrypsin. A novel biomarker for obesity in humans. Saudi Med J.

[49] Mendelson MM, Marioni RE, Joehanes R, Liu C, Hedman Å, Aslibekyan S, Demerath EW, Guan W, Zhi D, Yao C (2017). Association of body mass index with DNA methylation and gene expression in blood cells and relations to cardiometabolic disease: a Mendelian randomization approach. PLoS Med.

