# Supplementary material for: Cord blood DNA methylation and adiposity measures in early and mid-childhood
Source: Clin Epigenetics. 2017 Aug 15;9:86. doi: 10.1186/s13148-017-0384-9 (PMC5558655; doi:10.1186/s13148-017-0384-9)
Supplement: Additional file 1: — Online supplemental tables. (DOCX 18 kb) [file 13148_2017_384_MOESM1_ESM.docx]

| Table S1. Associations between adiposity outcomes before and after adjustment for child birthweight. | | | | | | | | |
| --- | --- | --- | --- | --- | --- | --- | --- | --- |
|  | Modelled without birthweight as covariate | | | |  | Modelled with birthweight as covariate | | |
|  | CpG site | Coef. | CI | q-value |  | Coef. | CI | q-value |
| Early Childhood Measurements | | | | | | | | |
| SS:TR | cg11137145 | 0.097 | (0.062, 0.133) | 0.0219 |  | 0.097 | (0.062, 0.133) | 0.0217 |
|  | cg03352173 | -0.066 | (-0.09, -0.041) | 0.0271 |  | -0.066 | (-0.09, -0.041) | 0.0270 |
|  | cg00885918 | -0.152 | (-0.21, -0.094) | 0.0271 |  | -0.152 | (-0.21, -0.094) | 0.0270 |
|  | cg20624923 | 0.221 | (0.135, 0.307) | 0.0348 |  | 0.221 | (0.136, 0.306) | 0.0283 |
|  |  |  |  |  |  |  |  |  |
| SS + TR | cg09271157 | 0.207 | (0.130, 0.283) | 0.0405 |  | 0.207 | (0.131, 0.283) | 0.0351 |
|  |  |  |  |  |  |  |  |  |
| Mid-childhood Measurements | | | | | | | | |
| SS:TR | cg14974711 | 0.191 | (0.120, 0.262) | 0.0452 |  | 0.191 | (0.120, 0.261) | 0.0482 |
|  |  |  |  |  |  |  |  |  |
| SS + TR | No significant findings | | | |  | No significant findings | | |
| Tot fat mass index |  |  |  |  |  |  |  |  |
| Fat free mass index |  |  |  |  |  |  |  |  |
| Tot % fat |  |  |  |  |  |  |  |  |
| Truncal fat index |  |  |  |  |  |  |  |  |
| Abbreviations: Coef., Coefficient; CI, confidence interval; SS, subscapular; TR, tricep  Presenting only significant results at (FDR) *q* < 0.05. | | | | | | | | |

| Table S2. Associations between six identified CpG sites with childhood caliper measurements of adiposity | | | | | | | | | | | | |
| --- | --- | --- | --- | --- | --- | --- | --- | --- | --- | --- | --- | --- |
| SS + TR |  | Early childhood | | | | | |  | | | | Mid-childhood |
|  | Gene | Coef. | | CI |  | Coef. | | | CI | | | |
| CpG site |  |  | |  |  | |  | | |  | | |
| cg11137145 | KPRP | -0.027 | | (-0.069, 0.015) |  | | 0.014 | | | (-0.053, 0.082) | | |
| cg03352173 | SLC9A10 | 0.000 | | (-0.032, 0.032) |  | | -0.019 | | | (-0.060, 0.022) | | |
| cg00885918 | MYLK2 | 0.002 | | (-0.075, 0.078) |  | | -0.032 | | | (-0.131, 0.068) | | |
| cg20624923 | PRLHR | -0.031 | | (-0.139, 0.078) |  | | 0.074 | | | (-0.110, 0.258) | | |
| cg09271157 | PPAPDC1A | **0.207** | | **(0.130, 0.283)** |  | | *0.129* | | | *(0.001, 0.257)* | | |
| cg14974711 | MMP25 | 0.071 | | (-0.014, 0.156) |  | | 0.052 | | | (-0.061, 0.166) | | |
|  |  |  | |  |  | |  | | |  | | |
| SS:TR |  |  | Mid-childhood | | | | | | | |  |  |
|  | Gene | Coef. | | CI |  | | Coef. | | | CI | | |
| CpG Site |  |  | |  |  | |  | | |  | | |
| cg11137145 | KPRP | **0.097** | | **(0.062, 0.133)** |  | | 0.015 | | | (-0.052, 0.083) | | |
| cg03352173 | SLC9A10 | **-0.066** | | **(-0.09, -0.041)** |  | | -0.014 | | | (-0.046, 0.019) | | |
| cg00885918 | MYLK2 | **-0.152** | | **(-0.21, -0.094)** |  | | -0.042 | | | (-0.114, 0.030) | | |
| cg20624923 | PRLHR | **0.221** | | **(0.135, 0.307)** |  | | -0.039 | | | (-0.159, 0.081) | | |
| cg09271157 | PPAPDC1A | -0.005 | | (-0.089, 0.078) |  | | 0.081 | | | (-0.005, 0.168) | | |
| cg14974711 | MMP25 | 0.033 | | (-0.039, 0.105) |  | | **0.191** | | | **(0.120, 0.262)** | | |
| Abbreviations: Coef., Coefficient; CI, confidence interval; SS, subscapular; TR, tricep  Bold denotes significant at *q* < 0.05, italics denotes significance at *P* < 0.05 | | | | | | | | | | | | |
